# The blood pressure variability in patients with cryptogenic stroke

**DOI:** 10.1186/s43044-022-00305-6

**Published:** 2022-09-24

**Authors:** Ahmed Alaarag, Hazem Abdelkhalek, Osama Amin

**Affiliations:** 1 Cardiology Department, Faculty of Medicine, Tanta University, Tanta, Egypt; 2 Neurology Department, Faculty of Medicine, Tanta University, Tanta, Egypt; 3 Cardiology Department, Faculty of Medicine, Beni-Suef University, Beni Suef, Egypt

**Keywords:** Cryptogenic stroke, Left atrial remodeling, Blood pressure variability

## Abstract

**Background:**

Increased nighttime BP variability (BPV) was associated with stroke. Left atrial (LA) enlargement is the default clinical hallmark of structural remodeling that often occurs in response to LA pressure and volume overload. Blood pressure has proven to be an essential determinant of LA enlargement. We aimed to evaluate the influence of BPV as a risk factor for cryptogenic stroke and highlight the importance of including the (APBM) in the workup for those patients and test the relation between BPV and LA remodeling in these patients, which could be used as a clue to add APM monitoring to their workup. Also, LA remodeling may be a substrate for occult atrial fibrillation (AF). We included Group I (108 consecutive patients with cryptogenic ischemic stroke) and Group II (100 consecutive adult participants without a history of stroke or any structural heart disease). We measured the maximal LA volume index (Max LAVI) and minimal LA volume index (Min LAVI). We calculated the left atrial ejection fraction (LAEF). All the participants were subjected to ABPM.

**Results:**

In our prospective, cross-sectional cohort study, the patients in Group I had statistically significantly higher Min LAVI and Max LAVI and Less LA EF than Group II, with a P value of (0.001, 0.001, and 0.008), respectively. The Group I patients had higher BPV as measured by SD parameters than patients in Group II, with a *P *value of 0.001 for all SD parameters. The BPV parameters, as measured by SD parameters, were positively related to the LA remodeling parameters in both groups. After adjusting all variables, we found that age, night systolic SD, and night diastolic SD parameters were independent predictors of LA remodeling.

**Conclusions:**

The patients with cryptogenic stroke had higher short-term BPV, Min LAVI, and Max LAVI but lower LA EF. Careful monitoring of BPV may be of value for both primary and secondary preventions of ischemic stroke.

## Background

Left atrial (LA) enlargement is frequently the default clinical hallmark of structural remodeling in response to LA pressure and volume overload [1]. Blood pressure (BP) has been a crucial determining factor of LA enlargement [2]. Conventional single BP measurement cannot reflect the patient’s intrinsic BP characteristics because of insufficient sampling and the probability of falsely higher reading due to emotional factors, such as the white coat impact [3].

A complementary method is Ambulatory Blood Pressure Monitoring (ABPM), which permits various measurements under more controlled and consistent conditions. Additionally, various BP measurements offer data about variability. Augmented nighttime BP variability (BPV) was associated with atherosclerosis and consequent stroke [3, 4]. The higher nighttime BP and its variability are independently related to LA enlargement [5]. The LA enlargement is a subclinical deviation that conveys a more significant stroke hazard [6].

Asymptomatic paroxysmal atrial fibrillation (AF) has been claimed to cause a cryptogenic stroke [7]. Loss of LA longitudinal deformation, decreased LAEF, and other structural LA remodeling and electrophysiological parameters were related to an augmented risk of paroxysmal AF in patients with cryptogenic stroke [8, 9].

Intensive intervention for these patients may delay LA remodeling progression and reduce the risk of ischemic stroke. However, the association between BPV and LA enlargement has not been addressed in patients with cryptogenic stroke. The current study aimed to evaluate the influence of BPV as a risk factor for cryptogenic stroke and to test the relation between BPV and LA remodeling in these patients, which could be used as a clue to add ABPM to their workup.

## Methods

This prospective, cross-sectional cohort study was performed from September 2019 to November 2021. The local research committees approved the study following the Declaration of Helsinki. We acquired informed written consent from all the participants. We included 108 consecutive patients with cryptogenic ischemic stroke (Group I). The stroke unit referred them for cardiac evaluation after suffering from a cerebral stroke.

We defined cryptogenic stroke as any ischemic stroke without apparent cause despite extensive history and corresponding studies. We subjected all the patients to a detailed history, including preceding events, such as recent invasive procedures, intravenous drug abuse, and recent pregnancy. We focused on atherosclerotic risk factors, including hypertension, dyslipidemia, and diabetes mellitus (DM). We reviewed the family history of premature atherosclerotic disease, cerebrovascular stroke (CVS), or sudden death. Then, we subjected all the patients to a detailed physical examination.

**Fig. 1:**
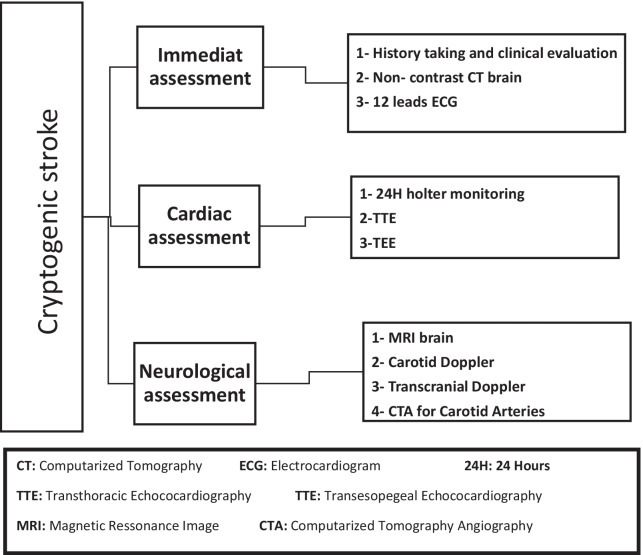
Workup for patients with cryptogenic stroke

The cardiac evaluation included a 12-lead electrocardiogram (ECG), ambulatory 24 h ECG to exclude AF, ABPM, transthoracic, and transesophageal echocardiography (TEE) to exclude a thrombus, cardiac mass, vegetation, and intracardiac shunt. We performed non-contrast CT brain, carotid ultrasound, transcranial Doppler, MRI brain, and CT angiography of the head and neck for all the patients to exclude patients with any apparent cause of stroke. We outlined the evaluation plan for cryptogenic stroke in Fig. 1.

All the included patients had sinus rhythm at the examination and left ventricular ejection fraction (LVEF) over 50%. According to the European Society of Echocardiography, we evaluated cardiac chamber size, LVEF, and LA dimensions [10]. We measured LV end-diastolic indexed volume (mL/m^2^) and LV end-systolic indexed volume (mL/m^2^) and also peak velocity of E and A waves of mitral flow, é wave was measured from the apical four-chamber view, with 2–5 mm sample volume taken from the septal corner of the mitral annulus, and E/é ratio was calculated.

We obtained the 2D volume of the LA from the apical view during a short breath-hold. We acquired 2D LA images from apical four- and two-chamber views. In 2D, we assessed the LA area on the four-chamber view in end systole. We measured volumes using the biplane modified Simpson’s method. We indexed LA volumes to the body surface area. We measured the maximal LA volume index (Max LAVI) at the ventricular end-systolic frame just before the mitral valve opening from the apical views. We measured minimal LA volume (Min LAVI) at the end of LV diastole, just before the mitral valve closure. We calculated the LA function with the following formula: LA ejection fraction (LAEF): (Max LAVI-Min LAVI)/Max LAVI × 100.

We excluded patients with atrial flutter and fibrillation, bundle branch block, poor acoustic window, and a history of coronary artery disease (CAD). Also, we excluded patients with previous percutaneous coronary intervention (PCI) or open-heart surgery, previous ablation procedure, pulmonary hypertension, and left ventricular hypertrophy (LVH). The ECG assessment included P-wave amplitude, QRS duration, and QT interval.

We performed a routine laboratory test, including the lipid profile and blood glucose level. We defined DM as a fasting plasma glucose > 140 mg/dl or the use of antidiabetic drugs. We described obesity as body mass index (BMI) ≥ 30 kg/m^2^. We defined hypertension according to the European Society of Cardiology Guidelines as systolic BP (SBP) ≥ 140 mmHg or diastolic BP (DBP) ≥ 90 mmHg [11]. We defined ABPM limits above the normal if the mean 24 h SBP was > 130 or DBP ≥ 80, the daytime was ≥ 135/ ≥ 85 mmHg, or the nighttime was ≥ 120/ ≥ 70 [12].

We defined hypercholesterolemia as total serum cholesterol > 220 mg/dl or the use of lipid-lowering medications. We informed the patients that cuff inflation might be associated with some distress. We used an ABPM device validated against accepted universal criteria, CONTEC 50, for all the patients. The data included all daytime and nighttime BP readings with an indication of normal BP, average SBP, DBP, and heart rate, time‐weighted average SBP, DBP, and heart rate for the 24 h, daytime, and nighttime, with standard deviations (SD) and the number of accurate BP readings, the percentage decline in nocturnal SBP and DBP. We used the non‐dominant upper limb for measurements and used a suitable cuff. We set the device to obtain BP readings every 15 min during the daytime (06:00–23:00 h) and every thirty minutes at night (23:00–06:00 h). Then, we confirmed that ≥ 70% of the expected 24‐h readings were valid and registered the ABPM data during regular workdays. We used the SD indices for the assessment of the short-term BPV.

Group II included 100 consecutive individuals who attended echocardiographic units without any history of cerebrovascular insult, cardiac ablation procedure, or overt structural heart disease (the same echocardiographic exclusion criteria as Group I). All were subjected to ABPM and echocardiographic measurements like patients in Group I.

### Statistical analysis

We analyzed the data using the Statistical Program for Social Science (SPSS) version 20.0. We expressed the quantitative data as mean ± SD. We described the qualitative data as frequency and percentage. We used the Kolmogorov–Smirnov test to analyze the normal distribution of continuous data, which was normally distributed.

We used an independent-samples t test of significance to compare the two means. We compared the means of age, 24H systolic SD, day systolic SD, night systolic SD, 24H diastolic SD, day diastolic SD, night diastolic SD, LVESVI mL/m^2^, LVEDVI mL/m^2^, Min LAVI mL/m^2^, Max LAVI mL/m^2^, and LA EF %.

We applied the chi-square (*X*^2^) test of significance to evaluate proportions between two qualitative parameters (risk factors and medications).

We used Pearson’s correlation coefficient (*r*) to test the LA remodeling parameters and their quantitative predictors. We used Spearman’s correlation coefficient (*r*) test for correlating the LA remodeling parameters and their qualitative predictors. We used the logistic multivariate regression analysis to study the multiple variables that may affect the LA remodeling parameters and to adjust the different potential confounders, including the basal demographic characteristics and the ABPM parameters. We presented the adjusted odds ratio with 95% confidence intervals.

We calculated the sample size and the power analysis using g*power software 3.1.9.4. We used the sample size calculation criteria: 95% confidence limit and 80% study power for different variables, and we found it at 100 patients. The cutoff point of statistical significance was 0.05.

We assessed intra-observer and inter-observer variability for echocardiographic parameters by randomly selecting 15 patients and repeating the analysis on the same cine loop by the same investigator or independently by two separate investigators using intraclass correlation coefficients (ICC) with values less than 0.5 indicating poor reliability, between 0.5 and 0.75 mean moderate reliability, between 0.75 and 0.9 mean good reliability, and above 0.9 excellent reliability.

#### Reproducibility

Intra-observer and inter-observer variability for echocardiographic parameters ranged from 0.91 and 0.95.

## Results

Our two-center comparative cross-sectional study included 108 consecutive patients with cryptogenic stroke in Group I and 100 patients without stroke in Group II. We found no significant difference between both groups regarding the basal demographic characteristics, risk factors, and antihypertensive medications, as shown in Tables 1 and 2.

**Table 1:** Comparing demographic characteristics of the study population

	Range	Mean ± SD	*t* test	*p* value
Age				
Group I	37–67	54.09 ± 7.04	0.747	0.388
Group II	39–68	53.21 ± 7.68		
Enrollment office SBP				
Group I	110–155	132.69 ± 13.88	0.069	0.945
Group II	110–155	132.55 ± 14.20		
Enrollment office DBP				
Group I	65–95	80.23 ± 8.24	0.805	0.422
Group II	65–95	81.15 ± 8.19		

		Group I	Group II	*X*^2^	*P* value
Gender					
Male	N	67	60	0.091	0.763
	%	62.0%	60.0%		
Female	N	41	40		
	%	38.0%	40.0%		
Smoking					
Yes	N	31	25	0.362	0.547
	%	28.7%	25.0%		
No	N	77	75		
	%	71.3%	75.0%		
Diabetes					
Yes	N	25	20	0.304	0.582
	%	23.1%	20.0%		
No	N	83	80		
	%	76.9%	80.0%		
Dyslipidemia					
Yes	N	22	17	0.387	0.534
	%	20.4%	17.0%		
No	N	86	83		
	%	79.6%	83.0%		
Hypertension					
Yes	N	37	28	0.947	0.331
	%	34.3%	28.0%		
No	N	71	72		
	%	65.7%	72.0%		
ABP measurements above limits of normal					
Yes	N	25	25	0.098	0.775
	%	23.1%	25.0%		
No	N	83	75		
	%	76.9%	75.0%		

SD, standard deviation; SBP, systolic blood pressure; DBP, diastolic blood pressure; and ABP, ambulatory blood pressure

**Table 2:** Comparing antihypertensive medication of the study population

		Group I	Group II	*X*^2^	*P* value
ACEIs					
Yes	N	16	11	0.669	0.413
	%	14.8%	11.0%		
No	N	92	89		
	%	85.2%	89.0%		
ARBs					
Yes	N	8	7	0.013	0.910
	%	7.4%	7.0%		
No	N	100	93		
	%	92.6%	93.0%		
BB					
Yes	N	23	14	1.890	0.169
	%	21.3%	14.0%		
No	N	85	86		
	%	78.7%	86.0%		
CCB					
Yes	N	9	9	0.029	0.864
	%	8.3%	9.0%		
No	N	99	91		
	%	91.7%	91.0%		
Diuretics					
Yes	N	19	10	2.495	0.114
	%	17.6%	10.0%		
No	N	89	90		
	%	82.4%	90.0%		

ACEIs, angiotensin-converting enzyme inhibitors; BB, beta-blocker; ARBs, angiotensin receptor blockers; and CCB, calcium channel blocker

In the cryptogenic group, 9.2% (*n* = 10) of patients had palpitation despite that ambulatory ECG monitoring did not detect any arrhythmias.

Moreover, there were no statistically significant variances between both groups in the left ventricular volumes ( LVESVI and LVEDVI), with P values of (0.867 and 0.956), respectively. There were no significant differences in the parameters that assess LV filling pressure and diastolic functions (peak E velocity, peak A velocity, and *E*/*é* ratio) with P values of (0.781, 0.926, and 0.409), respectively, as shown in Table 3.

**Table 3:** Comparing echocardiographic parameters and BPV parameters in both groups

	Range	Mean ± SD	*t* test	*p* value
24 H systolic SD				
Group I	9.8–17.9	13.29 ± 1.80	24.191	0.001*
Group II	9.6–16.4	12.11 ± 1.63		
Day systolic SD				
Group I	9.3–16.9	12.83 ± 1.75	23.562	0.001*
Group II	9.3–15.7	11.68 ± 1.64		
Night systolic SD				
Group I	8.9–15.1	11.99 ± 1.62	23.947	0.001*
Group II	8.9–14.9	10.93 ± 1.50		
24 H diastolic SD				
Group I	7.6–13.2	10.39 ± 1.31	23.712	0.001*
Group II	7.6–12.8	9.53 ± 1.21		
Day diastolic SD				
Group I	7.3–12.6	9.98 ± 1.27	22.460	0.001*
Group II	7.3–12.1	9.17 ± 1.19		
Night diastolic SD				
Group I	6.5–11.7	9.03 ± 1.28	19.733	0.001*
Group II	6.5–11.5	8.27 ± 1.17		
LVESVI (mL/m^2^)				
Group I	11–21	16.03 ± 2.52	0.028	0.867
Group II	11–21	15.97 ± 2.46		
LVEDVI (mL/m^2^)				
Group I	37–55	45.35 ± 3.77	0.003	0.956
Group II	37–55	45.32 ± 3.65		
Min LAVI (mL/m^2^)				
Group I	10–34	23.44 ± 5.94	73.710	0.001*
Group II	8–31	16.49 ± 5.71		
Max LAVI (mL/m^2^)				
Group I	29–55	43.69 ± 6.69	172.531	0.001*
Group II	22–49	32.51 ± 5.47		
LA EF %				
Group I	37–66	47.23 ± 6.29	7.225	0.008*
Group II	34–68	50.36 ± 10.17		
Peak E velocity (m/s)				
Group I	0.6–0.9	0.73 ± 0.11	0.279	0.781
Group II	0.6–0.9	0.73 ± 0.11		
Peak A velocity (m/s)				
Group I	0.6–1	0.78 ± 0.10	0.092	0.926
Group II	0.6–1	0.78 ± 0.11		
*E*/*é* ratio				
Group I	9–16	11.67 ± 1.75	0.827	0.409
Group II	9–16	11.47 ± 1.67		

LA, left atrial; BPV, blood pressure variability; SD, standard deviation; 24 H, 24 hours; LVESVI, left ventricular end-systolic volume index; LVEDVI, left ventricular end-diastolic volume index; Min LAVI, minimum left atrial volume index; Max LAVI, maximum left atrial volume index; and LA EF, left atrial ejection fraction

*Significant *P* value

While, regarding LA and BPV parameters, patients with cryptogenic stroke (Group I) had significantly higher Min LAVI and Max LAVI than Group II, with a *P* value of 0.001 for both parameters, simultaneously, patients with cryptogenic stroke had significantly lower LA EF, with a *P* value of 0.008, as shown in Table 3.

Also, patients with cryptogenic stroke had higher BPV as measured by SD parameters than Group II, with a *P* value of 0.001 for all SD parameters, as shown in Table 3.

We correlated the LA remodeling parameters to the different potential confounders, as shown in Tables 4 and 5. We found that aging and all BPV parameters, as measured by SD, were positively correlated with the (Min LAVI, Max LAVI). In contrast, both aging and BPV parameters were correlated negatively with the LA EF; this occurred in both groups. Finally, in cryptogenic stroke patients, we found a negative correlation between the *E*/*é* ratio and LA EF with a *P* value of 0.018.

**Table 4:** Correlation between echocardiographic parameters and the demographic data in Group I

Group I	Min LAVI mL/m^2^		Max LAVI mL/m^2^		LA EF %	
	*r*	*p*	*r*	*p*	*r*	*p*
24 H systolic SD	0.875	0.001*	0.870	0.001*	− 0.821	0.001*
Day systolic SD	0.258	0.007*	0.272	0.004*	− 0.224	0.020*
Night systolic SD	0.874	0.001*	0.873	0.001*	− 0.824	0.001*
24 H diastolic SD	0.864	0.001*	0.863	0.001*	− 0.808	0.001*
Day diastolic SD	0.853	0.001*	0.856	0.001*	− 0.800	0.001*
Night diastolic SD	0.805	0.001*	0.806	0.001*	− 0.767	0.001*
Peak E velocity (m/s)	− 0.058	0.552	− .017	0.857	0.118	0.223
Peak A velocity (m/s)	− .102	0.293	− 0.079	0.415	0.169	0.081
*E*/*é* ratio	− 0.033	0.737	− 0.030	0.759	− 0.227	0.018*
Enrollment office systolic BP	− 0.007	0.943	− 0.025	0.797	0.021	0.828
Enrollment office diastolic BP	− 0.035	0.723	− 0.041	0.673	0.063	0.518
ABP above limits of normal	0.003	0.973	0.030	0.757	0.020	0.835
Age	0.316	0.001*	0.313	0.001*	− 0.341	0.001*
Gender	− 0.060	0.534	− 0.105	0.281	0.029	0.765
Smoking	− 0.107	0.271	− 0.109	0.263	0.102	0.295
Diabetes	0.032	0.744	0.008	0.934	− 0.055	0.574
Dyslipidemia	− 0.018	0.852	0.001	0.999	0.034	0.727
Hypertension	− 0.024	0.802	− 0.031	0.747	0.003	0.972

24 H, 24 hours; LA, left atrial; LVESVI, left ventricular end-systolic volume index; BP, blood pressure; ABP, ambulatory blood pressure; LVEDVI, left ventricular end-diastolic volume index; Min LAVI, minimum left atrial volume index; Max LAVI, maximum left atrial volume index; LA EF, left atrial ejection fraction; and SD, standard deviation

*Significant *P* value

**Table 5:** Correlation between echocardiographic parameters and the demographic data in Group II

Group II	Min LAVI (mL/m^2^)		Max LAVI (mL/m^2^)		LA EF (%)	
	*r*	*p*	*r*	*p*	*r*	*p*
24 H systolic SD	0.866	0.001*	0.854	0.001*	− 0.771	0.001*
Day systolic SD	0.867	0.001*	0.852	0.001*	− 0.778	0.001*
Night systolic SD	0.846	0.001*	0.826	0.001*	− 0.774	0.001*
24 H diastolic SD	0.862	0.001*	0.814	0.001*	− 0.826	0.001*
Day diastolic SD	0.848	0.001*	0.795	0.001*	− 0.822	0.001*
Night diastolic SD	0.808	0.001*	0.745	0.001*	− 0.792	0.001*
Peak E velocity (m/s)	− 0.024	0.812	− 0.055	0.585	− 0.012	0.906
Peak A velocity (m/s)	0.034	0.735	− 0.006	0.956	− 0.074	0.469
*E*/*é* ratio	− 0.052	0.608	− 0.104	0.304	− 0.016	0.872
Enrollment office systolic BP	0.123	0.222	0.172	0.088	− 0.062	0.542
Enrollment office diastolic BP	− 0.047	0.644	0.010	0.918	0.127	0.212
ABP measurements above limits of normal	− 0.190	0.058	− 0.101	0.145	0.186	0.066
Age	0.457	0.001*	0.454	0.001*	− 0.389	0.001*
Gender	− 0.021	0.836	− 0.032	0.750	− 0.008	0.941
Smoking	0.003	0.978	0.003	0.975	0.015	0.879
Diabetes	− 0.051	0.613	− 0.025	0.803	0.039	0.703
Dyslipidemia	0.072	0.476	0.018	0.859	− 0.098	0.337
Hypertension	0.060	0.556	0.057	0.574	− 0.043	0.673

24 H, 24 hours; LA, left atrial; LVESVI, left ventricular end-systolic volume index; BP, blood pressure; ABP, ambulatory blood pressure; LVEDVI, left ventricular end-diastolic volume index; Min LAVI, minimum left atrial volume index; Max LAVI, maximum left atrial volume index; LA EF, left atrial ejection fraction; and SD, standard deviation

*Significant *P* value

We did a logistic multivariate regression analysis for the covariant. It was significantly correlated with the LA remodeling parameters (Min LAVI, Max LAVI, and LA EF). After adjusting all variables, age, night systolic SD, and night diastolic SD parameters were independent predictors of LA remodeling, as shown in Table 6.

**Table 6:** Multivariate regression analysis for covariants affecting the LA remodeling parameters

	Min LAVI		Max LAVI		LA EF %	
	OR (95% CI)	*P* value	OR (95% CI)	*P* value	OR (95% CI)	*P* value
Age	0.532 (0.148–0.653)	0.008*	0.628 (0.254–0.861)	0.011*	1.625 (1.068–5.219)	0.012*
24 H systolic SD	0.704 (0.245–2.025)	0.515	0.385 (0.248–3.526)	0.628	2.637 (0.659–6.952)	0.515
Day systolic SD	0.945 (0.402–2.221)	0.397	0.754 (0.394–4.921)	0.423	2.531 (0.953–3.627)	0.397
Night systolic SD	0.364 (0.097–0.483)	0.003*	0.469 (0.109–0.652)	0.006*	1.637 (1.203–4.526)	0.008*
24 H diastolic SD	0.611 (0.138–2.696)	0.467	0.574 (0.235–1.958)	0.523	1.362 (0.583–3.627)	0.467
Day diastolic SD	0.617 (0.189–5.215)	0.509	0.845 (0.539–9.652)	0.509	2.657 (0.567–8.529)	0.509
Night diastolic SD	0.462 (0.304–0.785)	0.001*	0.529 (0.258–0.951)	0.001*	2.315 (1.583–5.326)	0.001*
*E*/*é* ratio	0.524 (0.412–2.321)	0.327	0.472 (0.318–1.746)	0.451	1.854 (0.957–4.531)	0.092

Min LAVI, minimum left atrial volume index; LA, left atrial; Max LAVI, maximum left atrial volume index; LA EF, left atrial ejection fraction; 24 H, 24 hours; CI, confidence interval; and OR, odds ratio

*Significant *P* value

These results could propose that high BPV in patients with cryptogenic stroke may lead to abnormal LA remodeling and function, which may be a substrate for occult AF, the source of stroke in these patients.

## Discussion

Stroke was the fifth principal reason for death, and cryptogenic stroke represents 15–40% of ischemic strokes in different studies [13]. The recent guidelines do not make specific recommendations to prevent cryptogenic stroke [14].

In our study, the high BPV was associated with structural and functional LA remodeling. Moreover, structural and functional LA remodeling was more prevalent in patients with cryptogenic stroke. Consequently, the high BPV may lead to LA structural and functional changes and an increased risk of cryptogenic stroke. The results were similar to Vural MG et al. [15], who found a relation between LA remodeling and cryptogenic stroke in young patients.

LA remodeling denotes the spectrum of pathophysiological alterations in the LA mechanical and electrical function that respond to stress disorders such as hypertension, heart failure, DM, and obesity. Structural LA remodeling often responds to LA pressure and volume overload [1, 16].

Remodeling is primarily adaptive, but it often becomes maladaptive in response to a chronic pathological provocation, and it is linked with an enhanced probability of cardiovascular morbidity and mortality [16]. In our study, we focused on mechanical remodeling.

We used Min LAVI, Max LAVI, and LA EF to study maladaptive mechanical remodeling. We found that patients with cryptogenic stroke had statistically significantly lower LA EF than Group II. Chinali M et al. [17] studied about 2800 patients with a high prevalence of obesity, DM, and LA systolic dysfunction and found a higher rate of cardiovascular events.

Some studies found that reduced LA EF was associated with increased pressure in the LA appendage and abnormal LA strain. This strain may be a substrate for thromboembolism [15, 18].

Norioka et al. [5] studied the impact of BPV on LA function and structure in 140 patients with paroxysmal AF and normal EF. They concluded that higher nighttime BP and its variability were associated with LA enlargement, and nighttime BP and its variability have additional predictive value for LA remodeling. Moreover, Doménech et al. found that nighttime BP was associated with LA size and the release of natriuretic peptides in patients with idiopathic AF [19].

High BPV was strongly associated with LA remodeling and reduced LA EF in our study. So, more intensive investigation and more careful evaluation may be required for patients with cryptogenic stroke who have LA remodeling, which may be a clue for high BPV or a substrate for occult atrial fibrillation with increased risk of stroke in our study. 9.2% of patients in the cryptogenic group had symptoms of palpitation despite that Holter monitoring did not diagnose any arrhythmias.

The limitations of this study include the relatively small number of patients. Also, both left atrial volumes and LA EF are affected by multiple factors.

## Conclusions

The patients with cryptogenic stroke had higher short-term BPV as measured by SD. Also, the patients with cryptogenic stroke had higher Min LAVI, Max LAVI, and lower LA EF. LA remodeling may be a substrate for paroxysmal AF and subsequent thrombo-embolization, and these patients may require heart rate monitoring for a more extended period to detect arrhythmias. Also, the presence of left atrial remodeling in echocardiographic screening for patients with cryptogenic stroke may be a clue to adding the APBM to the workup. Tackling BPV may be of value for both primary and secondary preventions of cryptogenic stroke.

## Data Availability

The datasets used and or analyzed during the current study are available from the corresponding author upon request.

## Abbreviations

LA: Left atrial
BP: Blood pressure
LVEF: Left ventricular ejection fraction
Max LAVI: Maximal LA Volume Index
Min LAVI: Minimal LA Volume Index
LAEF: LA ejection fraction
SBP: Systolic blood pressure
DBP: Diastolic blood pressure
CT: Computerized tomography
MRI: Magnetic resonance imaging
AF: Atrial fibrillation
SD: Standard deviation
LVESVI: Left ventricular end-systolic volume index
LVEDVI: Left ventricular end-diastolic volume index

## References

[1] Tsang TS, Barnes ME, Gersh BJ, Bailey KR, Seward JB (2002). Left atrial volume as a morphophysiologic expression of left ventricular diastolic dysfunction and relation to cardiovascular risk burden. Am J Cardiol.

[2] McManus DD, Xanthakis V, Sullivan LM (2010). Longitudinal tracking of left atrial diameter over the adult life course: clinical correlates in the community. Circulation.

[3] Palatini P, Reboldi G, Beilin LJ (2014). Added predictive value of nighttime blood pressure variability for cardiovascular events and mortality: the Ambulatory Blood Pressure-International Study. Hypertension.

[4] Iwata S, Sugioka K, Fujita S (2015). Aortic arch atherosclerosis in patients with severe aortic stenosis can be argued by greater day-by-day blood pressure variability. Atherosclerosis.

[5] Norioka N, Iwata S, Ito A (2018). Greater nighttime blood pressure variability is associated with left atrial enlargement in atrial fibrillation patients with preserved ejection fraction. Hypertens Res.

[6] Di Tullio MR, Sacco RL, Sciacca RR, Homma S (1999). Left atrial size and the risk of ischemic stroke in an ethnically mixed population. Stroke.

[7] Flint AC, Banki NM, Ren X, Rao VA, Go AS (2012). Detection of paroxysmal atrial fibrillation by 30-day event monitoring in cryptogenic ischemic stroke: the Stroke and Monitoring for paroxysmal AF in Real-Time (SMART) Registry. Stroke.

[8] Motoki H, Negishi K, Kusunose K, Popović ZB, Bhargava M, Wazni OM (2014). Global left atrial strain in the prediction of si¬nus rhythm maintenance after catheter ablation for atrial fibril¬lation. J Am Soc Echocardiogr.

[9] Na JO, Shin SY, Lim HE, Choi CU, Kim SH, Kim JW (2011). Impaired transport function of the left atrium and left atrial appendage in cryptogenic stroke patients with atrial septal aneurysm and without patent foramen ovale. Eur J Echocardiogr.

[10] Lang RM, Badano LP, Mor-Avi V (2015). Recommendations for cardiac chamber quantification by echocardiography in adults: an update from the American Society of Echocardiography and the European Association of Cardiovascular Imaging. J Am Soc Echocardiogr..

[11] Williams B, Mancia G, Spiering W (2018). 2018 ESC/ESH guidelines for the management of arterial hypertension. Eur Heart J..

[12] Volpe M, Gallo G, Battistoni A, Tocci G (2019). Highlights of ESC/ESH 2018 guidelines on the management of hypertension: what every doctor should know. High Blood Press Cardiovasc Prev.

[13] Benjamin EJ, Muntner P, Alonso A (2019). Heart disease and stroke statistics-2019 update: a report from the American Heart Association. Circulation..

[14] Kleindorfer DO, Towfighi A, Chaturvedi S (2021). 2021 Guideline for the prevention of stroke in patients with stroke and transient ischemic attack: a guideline from the American Heart Association/American Stroke Association. Stroke..

[15] Vural MG, Cetin S, Yilmaz M, Akdemir R, Gunduz H (2015). Relation between left atrial remodeling in young patients with cryptogenic stroke and normal inter-atrial anatomy. J Stroke.

[16] Hoit BD (2014). Left atrial size and function: role in prognosis. J Am Coll Cardiol.

[17] Chinali M, de Simone G, Roman MJ (2005). Left atrial systolic force and cardiovascular outcome: the strong heart study. Am J Hypertens..

[18] Goch A, Banach M, Piotrowski G, Szadkowska I, Goch JH (2007). Echocardiographic evaluation of the left atrium and left atrial appendage function in patients with atrial septum aneurysm: implications for thromboembolic complications. Thorac Cardiovasc Surg.

[19] Doménech M, Berruezo A, Molina I, Mont L, Coca A (2013). Nighttime ambulatory blood pressure is associated with atrial remodelling and neurohormonal activation in patients with idiopathic atrial fibrillation. Rev Esp Cardiol (Engl Ed).

