# Correction: The blood pressure variability in patients with cryptogenic stroke

**DOI:** 10.1186/s43044-022-00313-6

**Published:** 2022-10-03

**Authors:** Ahmed Alaarag, Hazem Abdelkhalek, Osama Amin

**Affiliations:** 1 Cardiology Department, Faculty of Medicine, Tanta University, Tanta, Egypt; 2 Neurology Department, Faculty of Medicine, Tanta University, Tanta, Egypt; 3 Cardiology Department, Faculty of Medicine, Beni-Suef University, Beni Suef, Egypt

## Correction: The Egyptian Heart Journal (2022) 74:68 https://doi.org/10.1186/s43044-022-00305-6

Following publication of the original article [1], the authors identified an error in the author name of Hazem Abdelkhalek.

The incorrect author name is: Hazem Abdelkalel.

The correct author name is: Hazem Abdelkhalek.

The author group has been updated above, and the original article [1] has been corrected.
